# A Comparative Analysis of Methods (LC-MS/MS, LC-MS and Rapid Test Kits) for the Determination of Diarrhetic Shellfish Toxins in Oysters, Mussels and Pipis

**DOI:** 10.3390/toxins13080563

**Published:** 2021-08-11

**Authors:** Penelope A. Ajani, Chowdhury Sarowar, Alison Turnbull, Hazel Farrell, Anthony Zammit, Stuart Helleren, Gustaaf Hallegraeff, Shauna A. Murray

**Affiliations:** 1 School of Life Sciences, University of Technology Sydney, P.O. Box 123, Broadway, NSW 2007, Australia; Shauna.Murray@uts.edu.au; 2 Sydney Institute of Marine Science, 19 Chowder Bay Road, Mosman, NSW 2088, Australia; Chowdhury.Sarowar@sims.org.au; 3 Institute for Marine and Antarctic Science, University of Tasmania, 15-21 Nubeena Crescent, Taroona, TAS 7053, Australia; alison.turnbull@utas.edu.au (A.T.); gustaaf.hallegraeff@utas.edu.au (G.H.); 4 NSW Food Authority, NSW Department of Primary Industries, P.O. Box 232, Taree, NSW 2430, Australia; Hazel.Farrell@dpi.nsw.gov.au (H.F.); Anthony.Zammit@dpi.nsw.gov.au (A.Z.); 5 Dalcon Environmental, Building 38, 3 Baron-Hay Ct, South Perth, WA 6151, Australia; stuart.helleren@dalconenvironmental.com.au

**Keywords:** LC-MS, rapid test kit, biotoxins, shellfish, diarrhetic shellfish toxins, *Dinophysis*

## Abstract

Rapid methods for the detection of biotoxins in shellfish can assist the seafood industry and safeguard public health. Diarrhetic Shellfish Toxins (DSTs) are produced by species of the dinoflagellate genus *Dinophysis*, yet the comparative efficacy of their detection methods has not been systematically determined. Here, we examined DSTs in spiked and naturally contaminated shellfish–Sydney Rock Oysters (*Saccostrea glomerata*), Pacific Oysters (*Magallana gigas*/*Crassostrea gigas*), Blue Mussels (*Mytilus galloprovincialis*) and Pipis (*Plebidonax deltoides*/*Donax deltoides*), using LC-MS/MS and LC-MS in 4 laboratories, and 5 rapid test kits (quantitative Enzyme-Linked Immunosorbent Assay (ELISA) and Protein Phosphatase Inhibition Assay (PP2A), and qualitative Lateral Flow Assay (LFA)). We found all toxins in all species could be recovered by all laboratories using LC-MS/MS (Liquid Chromatography—tandem Mass Spectrometry) and LC-MS (Liquid Chromatography—Mass Spectrometry); however, DST recovery at low and mid-level concentrations (<0.1 mg/kg) was variable (0–150%), while recovery at high-level concentrations (>0.86 mg/kg) was higher (60–262%). While no clear differences were observed between shellfish, all kits delivered an unacceptably high level (25–100%) of falsely compliant results for spiked samples. The LFA and the PP2A kits performed satisfactorily for naturally contaminated pipis (0%, 5% falsely compliant, respectively). There were correlations between spiked DSTs and quantitative methods was highest for LC-MS (r^2^ = 0.86) and the PP2A kit (r^2^ = 0.72). Overall, our results do not support the use of any DST rapid test kit as a stand-alone quality assurance measure at this time.

## 1. Introduction

Marine biotoxins are toxic chemical compounds produced by certain microalgae, which can bioaccumulate in shellfish and other marine organisms, and cause poisoning to seafood consumers. As well as seafood related illnesses, marine biotoxin contamination can lead to damaged public perceptions of seafood, direct economic losses and a restriction in the growth of the shellfish industry.

Diarrhetic Shellfish Toxins (DSTs) are produced by dinoflagellates of the planktonic genus *Dinophysis* and *Phalacroma*, and more rarely benthic *Prorocentrum*, and can bioaccumulate in shellfish and cause Diarrhetic Shellfish Poisoning (DSP). With approximately 11,000 human poisonings reported globally over the period 1985–2018 [1], DSP is a gastrointestinal disorder caused by the human consumption of seafood contaminated with DSTs. While symptoms are dose dependent and include diarrhea, nausea, vomiting and abdominal pain, DSTs are potent inhibitors of certain protein phosphatases and may promote tumor/cancer formation [2], although the impact of chronic exposure to DSTs is still not well known.

DSTs are a group of heat stable, polyether toxins consisting of okadaic acid (OA) and its isomer 19-epi-okadaic acid; the OA congeners dinophysistoxin-1 (DTX-1) and dinophysistoxin-2 (DTX-2); and the 7-acyl derivatives of OA, DTX-1 and DTX-2 that are collectively known as DTX-3. Together, they are referred to as the OA group toxins or the ‘okadaates’ (OAs). While OA, DTX-1 and DTX-2 only differ slightly in their molecular structure, the DTX-3 (group) includes a wide range of derivatives esterified with saturated and unsaturated fatty acids, products of metabolic transformations that occur in the shellfish [3]. Chemical compounds of this group are therefore generally described as either ‘free’ (unesterified) or ‘esterified’ [4].

DSP was first described after a large toxin event occurred in Japan in 1976 [5,6], whereby many people became sick after eating scallops (*Patinopecten yessoensis*). This contamination was linked to toxins produced by *Dinophysis fortii*. Following this event, further toxic episodes occurred in Japan, Spain and France, with several thousands of cases of human poisonings occurring over the 1970s and 1980s, and leading to the development of many regional monitoring programs. This monitoring has seen a gradual increase in reported DSP episodes in countries including Chile, Argentina, Mexico, the east coast of North America, Scandinavia, Ireland, Great Britain, Spain, Portugal, Italy, Greece, India, Thailand, Australia and New Zealand [5,7,8,9].

*Dinophysis* is common in Australian waters, with 36 species reported [10,11,12]. Toxic species include *D. acuminata* Claparede and Lachmann, *D. acuta* Ehrenberg, *D. caudata* Saville-Kent, *D. fortii* Pavillard, *D. norvegica* Claparede and Lachmann and *D. tripos* Gourret. There have been three serious DSP events in Australia. The first episode was caused by contamination of Pipis (*Plebidonax deltoides*) in New South Wales in 1997 (NSW) by *D. acuminata* [13]. One hundred and two people were affected and 56 cases of gastroenteritis were reported. A second episode occurred again in NSW in March 1998, this time with 20 cases of DSP poisoning reported [14]. The final event occurred in Queensland in March 2000, which was again linked to the consumption of Pipis [15]. While no human fatalities from DSP are known globally, DSTs continue to be a major food safety challenge for the shellfish industry.

Detection methods for DSTs using liquid chromatography with tandem mass spectrometry (LC-MS/MS and LC-MS) [4,16] and implemented as part of seafood safety programs, are considered the “gold standard” across the globe. These methods replaced the mouse bioassay (MBA), which was previously the most commonly used laboratory analysis tool (e.g., [17]). However, the development of more rapid, cost effective (on farm) testing methods for the presence of DSTs would potentially make harvest management simpler and faster and result in fewer closures. Three types of rapid test kits for the detection of DSTs are currently commercially available. These include an antibody-based enzyme-linked immunosorbent assay (ELISA) test; a functional protein phosphatase inhibition activity (PPIA) assay; and a lateral flow analysis (LFA) rapid test. ELISA assays involve an antigen immobilized on a (micro) plate, which are then complexed with an antibody that is linked to a reporter enzyme. These assays were first developed in the 1960s and 1970s for primarily medical diagnosis purposes [18]. Detection of OA, DTX-1 and DTX-2 (varying analogue cross reactivity depending on kit) is accomplished by assessing the conjugated enzyme activity via incubation with a substrate to produce a quantifiable product. Functional PPIA assays quantify okadaic acid (OA) and DST analogues including DTX-1, DTX-2 and DTX3 by colorimetric phosphatase inhibition, based on the reversible inhibition of protein phosphatase type 2A (PP2A) by the toxin, and the resulting absorbance derived from enzymatic hydrolysis of the substrate. A lateral flow test involves the shellfish extract transported across a reagent zone in which OA, DTX-1, DTX-2 and DTX-3 specific antibodies are combined with colored particles. If a toxin is present, it is captured by the particle-antibody complex, and as its concentration increases, the intensity of the test “line” decreases [19].

In a comprehensive review by McLeod et al. [20] of the currently available field methods for detection of marine biotoxins in shellfish, it was concluded that the ELISAs and LFAs had poor reactivity to the DSP congener DTX-2 and can give false negative results when high levels of DTX-3 are present (and the hydrolysis step is not undertaken to release ester forms). LFAs were also found to give some false positive results when DSP was below the ML (Max Limit), but this was dependent on the toxin profile, geographic region and shellfish species involved. Pectenotoxins (PTXs) are not currently included in Codex Standard for Live and Raw Bivalve Molluscs [21], and therefore are not included in this study. Several other jurisdictions such as Canada, Chile and the European Union do regulate for PTX (but not PTX-2sa), but the European Food Safety Authority has issued an opinion to deregulate PTX [22]. Furthermore, DSP regulation in Australia is governed by Food Standards Australia New Zealand with a maximum regulatory limit of 0.2 mg OA eq/kg [23], while most international standards including the Codex Standard, state a ML of 0.16 mg OA eq/kg [21].

To date, these rapid detection kits have not been tested on various shellfish matrices in a systematic manner, nor a comparison made across multiple analytical laboratories to assess LC-MS/MS or LC-MS detection of DSTs in shellfish. With this in mind, the present study aimed to undertake a comparative study to detect DSTs in differing shellfish matrices using commonly implemented protocols for LC-MS/MS or LC-MS in several different laboratories, as well as compare five commercially available rapid test kits for the detection of DSTs in these same shellfish tissues. The rapid test kits included three quantitative ELISA kits by Beacon^TM^, Eurofins/Abraxis^TM^ and EuroProxima^TM^; a quantitative PP2A kit by Eurofins/Abraxis^TM^, and a qualitative LFA kit by Neogen^TM^.

## 2. Results

### 2.1. LC-MS/MS and LC-MS

No toxins were detected in any of the four shellfish species matrices (Sydney Rock Oysters (*Saccostrea glomerata*) (SRO), Pacific Oysters (*Magallana gigas*/*Crassostrea gigas*) (PO), Blue Mussels (*Mytilus galloprovincialis*) (MUS) and Pipis (*Plebidonax deltoides*/*Donax deltoides*) (PIPI)) screened before spiking began (see Methods). Of the triplicate SROs spiked with OA at 0.02 mg/kg, Laboratory 1 detected OA in all three samples (x = 0.01, SD ± 0.00, min <0.01, max 0.02 mg/kg), Laboratory 2 and 4 reported concentrations below the detection limit for all samples (<0.01 mg/kg and <0.025 mg/kg respectively), and Laboratory 3 detected OA in all three samples (x = 0.013, SD ± 0.006, min 0.01, max 0.02 mg/kg). In summary, two out of the four laboratories detected OA at this low level, with recoveries between ~50–100% (Table 1).

Of the four shellfish species spiked with OA at 0.02 mg/kg, Laboratory 1 detected this toxin in all four matrices (x = 0.013, SD ± 0.005, min 0.01, max 0.02 mg/kg), Laboratory 2 did not detect OA in SRO or PO; however, it was detected in both MUS and PIPI (x = 0.015, SD ± 0.007; min <0.01, max 0.02 mg/kg), and Laboratory 3 did not detect OA in PO or MUS, but detected it in SRO and PIPI (x = 0.015, SD ± 0.007; min <0.01, max 0.02 mg/kg). Laboratory 4 did not detect OA at this concentration (less than detection limit <0.025 mg/kg). Laboratory 4, however, did detect OA in one PIPI sample at 0.03 mg/kg (>spike concentration). In summary, OA was detected in all matrices at this concentration, although not all laboratories detected toxins in all four matrices. Recovery across all laboratories ranged from ~50–150% (Table 2).

For the shellfish spiked with DTX-1 at 0.04 mg/kg, Laboratory 1 recovered this analogue in all matrices (x = 0.035, SD ± 0.006; min 0.03, max 0.05 mg/kg), with one PIPI sample returning a concentration of 0.01 OA mg/kg. Laboratory 2 detected DTX-1 in all matrices (x = 0.025, SD ± 0.006; min 0.02, max 0.03 mg/kg), also with a detection of OA in PIPI at 0.02 mg/kg. Laboratory 3 detected DTX-1 in all matrices (x = 0.025, SD ± 0.006; min 0.02, max 0.03 mg/kg), while Laboratory 4 did not detected this toxin in MUS (other matrices x = 0.026, min <0.025, max 0.04 mg/kg) (Table 3). In summary, DTX-1 was detected in all shellfish matrices at this concentration; however, one laboratory did not detect DTX-1 in MUS. The overall recovery of this analogue was ~50–100% across laboratories with two detections of OA in PIPIs.

For all shellfish spiked with DTX-2 at 0.01 mg/kg, Laboratory 1 did not recover this analogue in SRO or PIPI, and was only detected it in PO and MUS (both at 0.01 mg/kg). No toxin at this concentration was recovered from either Laboratory 2 nor Laboratory 3, while Laboratory 4 was unable to detect this toxin (below the limit of reporting <0.025 mg/kg) (Table 4). In summary DTX-2 was only detected in PO and MUS at this low concentration, and only at one laboratory. Overall recovery was ~50–100%.

When shellfish were spiked with all toxins (in varying concentrations between 2–10 × LOR depending on toxin analogue; see Methods), laboratory recovery of total toxin per sample for each laboratory was as follows: Laboratory 1: 53–75%; Laboratory 2: 35–88%; Laboratory 3: 13–41%; and Laboratory 4: 0–88% (Table 5). More specifically, all toxins were recovered in all matrices for Laboratory 1, with an individual toxin/sample recovery ranging from 40–200%, with the lowest matrix average recovery in SRO at 57% and the highest in PIPI at 103%. For Laboratory 2, DTX-2 was not detected in SRO or PO, while individual toxin/sample recovery ranged from 40–400%, with the lowest matrix average recovery in SRO at 43%, and the highest in PIPI at 170%. For Laboratory 3, OA was not detected in MUS or PIPI, and DTX-2 was not detected in PIPI. The individual toxin/sample recovery ranged from 20–50%, with the lowest matrix average in PIPI at 40% and the highest in MUS at 47%. Finally, for Laboratory 4, DTX-2 was not detected across all matrices and OA was not detected in MUS. Individual toxin/sample recovery ranged from 50–340% with the lowest matrix average in MUS at 50% and the highest in PIPI at 154%. Overall, most toxins were detected by all laboratories at these concentrations, individual recovery across all labs/matrices ranged from 0–88%, while the recovery across shellfish matrices varied.

In our final analysis to determine the recovery of CRM (OA/DTX-1/DTX-2), all laboratories detected all toxin analogues. Individual toxin recoveries ranged from 88 to 131% for Laboratory 1, 79–81% for Laboratory 2, 83–95% for Laboratory 3 and 101–262% for Laboratory 4 (Table 6). However, considering that these recoveries are the result of one sample per lab, they should be treated as indicative only.

### 2.2. Rapid Test Kits

#### 2.2.1. Wild Harvest Pipis

Prior to rapid test kit screening, OA, DTX-1 and DTX-2 analysis by LC-MS for wild harvest Pipis resulted in a OA toxin range of 0.1 to 0.3 mg/kg (Sample 4A—0.1 mg/kg, 4B—0.1 mg/kg, 4C—0.2 mg/kg, and 4D—0.3 mg/kg). After hydrolysis, no DTX-1 or DTX-2 was detected in any samples. Three batches comprising 10 replicates of each OA toxin concentration of 0.1, 0.2 and 0.3 mg/kg were subsequently screened using each rapid test kit.

#### 2.2.2. LC-MS

**Figure 1:**
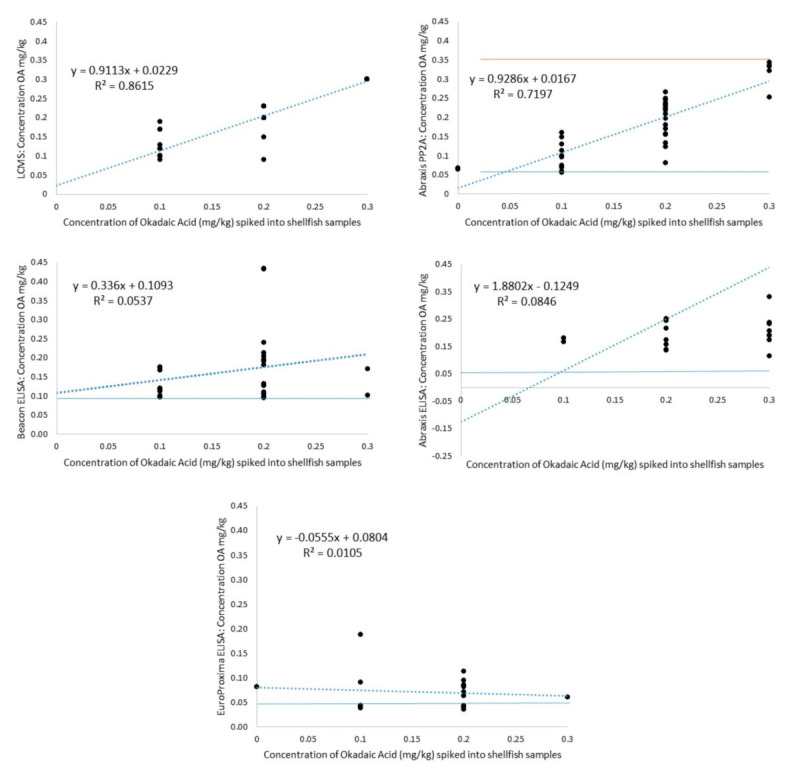
Linear regression plots showing the relationship between spiked toxin concentration with both LC-MS and quantitative rapid test kits results in Australian shellfish samples calculated data within each method’s working range. Blue lines represent lower working range and red line upper working range of method. Note: Abraxis PP2A Working Range (WR) = 0.06 to 0.35 mg/kg; Beacon ELISA Limit of Quantification (LOQ) = 0.1 mg/kg; Abraxis ELISA Working Range = 0.1–5.0 mg/kg; EuroProxima ELISA Limit of Quantification = 0.04 mg/kg.

Using LC-MS (Laboratory 3), all control shellfish samples (no toxin added) returned a ‘not detected’ result (Table 7). For OA spiked samples, 43/46 (~93%) returned concentrations at, or slightly above, the spiked toxin concentrations 0.1 and 0.2 mg/kg (Table 7 and Table 8). The three samples (7%) that returned concentrations lower that the spiked concentration were all spiked Pipi samples: sample 22 reported 0.09 mg/kg when it was spiked with OA at 0.1 mg/kg; sample 23 reported 0.15 mg/kg when it was spiked with OA at 0.2 mg/kg; and finally, sample 24 reported 0.09 mg/kg when it was spiked with OA at 0.2 mg/kg (Table 7 and Table 8). The latter two of these samples were falsely compliant at the regulatory limit (7%, 2/28). A Pearson’s correlation analysis between LC-MS results and the concentration of spiked toxin revealed a very strong relationship (r^2^ = 0.86) (Figure 1). Subsequently, this method returned a mean recovery of 106.5%, meeting the criteria set out in the AOAC Guidelines for Single Laboratory Validation of Chemical Methods for Dietary Supplements and Botanicals (AOAC 2002).

#### 2.2.3. Rapid Test Kits

##### Qualitative Test

##### Neogen

The Neogen kit returned negative readings for the eight negative control samples across all species-specific shellfish matrices. However, 23 out of 46 samples (50%) of spiked samples (across all shellfish matrices) returned a negative result when they contained okadaic acid (Table 7 and Table 8). Within this group, 18% (5/28 samples again across all matrices) returned a false compliant result when they were spiked at, or above, the regulatory limit (=/> 0.2 mg OA eq/kg), while no naturally contaminated Pipis returned falsely compliant results with this kit.

##### Quantitative Tests

##### Abraxis PP2A

The Abraxis PP2A returned 25% (2/8) false positive results, that is, they returned concentrations of toxin within the kit’s working (range 0.06 to 0.35 mg/kg), when the samples contained no okadaic acid. Of those shellfish that were spiked, 29% (13/45) of samples returned values that were outside the working range (8 samples below 0.06 mg/kg and 5 samples above 0.35 mg/kg), with 27% (12/45) samples being underestimated and 44% (20/45) returning a concentration which was equal to, or greater than, the spiked toxin concentration (Table 7 and Table 8). When samples were spiked at, or above, the regulatory limit, the Abraxis PP2A returned 29% (8/28) falsely compliant results (Table 9). These results were for both spiked and naturally contaminated samples. A Pearson’s correlation analysis between the Abraxis PP2A results and spiked toxin concentrations was significant at r^2^ = 0.72 (Figure 1). This kit returned a mean recovery of 92.2%, again meeting the criteria set out in the AOAC Guidelines [24] (Table 9).

##### Beacon ELISA

With a limit of quantification reported as 0.1 mg/kg, the Beacon ELISA kit returned 0% (0/8) false positives and 43% (20/46) of spiked samples below the limit of quantification. Of the samples that were spiked (and results above the quantification limit), 22% (10/46) were underestimated, while 35% (16/46) were equal to, or greater than, the spiked toxin concentration (Table 7 and Table 8). When samples were spiked at/above the regulatory limit, or were naturally contaminated at/above the regulatory limit, the Beacon ELISA returned 79% (22/28) falsely compliant results (Table 9). A Pearson’s correlation analysis between the Beacon ELISA kit test results and the spiked toxin concentrations was extremely weak at r^2^ = 0.05 (Figure 1). This kit returned a mean recovery of 77%, outside the criteria in the AOAC Guidelines [24] (Table 10).

##### Abraxis ELISA

Similar to the Abraxis PP2A, the Abraxis ELISA reports a working range of 0.01 to 0.5 mg/kg. This kit returned 0% (0/8) false positives and 59% (27/46) of spiked samples below the working range. Of the samples that were spiked (and results within the working range), 24% (11/46) were underestimated and 17% (8/46) were equal to, or greater than, the spiked toxin concentration (Table 7 and Table 8). Again, when spiked or naturally contaminated at/above the regulatory limit, the Abraxis ELISA returned 71% (20/28) falsely compliant results (Table 10). A Pearson’s correlation analysis between the Abraxis ELISA kit test results and the spiked toxin concentrations was weak at r^2^ = 0.08 (Figure 1). Subsequently, this kit returned a mean recovery of 66%, well outside the criteria in the AOAC Guidelines [24] (Table 10).

##### EuroProxima ELISA

With a limit of quantification reported as 0.04 mg/kg, the EuroProxima ELISA kit returned 13% (1/8) false positives and 65% (30/46) of spiked samples returning results outside the limit of quantification (<0.04 mg/kg). Of the samples that were spiked (and results reported were above the limit of quantification), 33% (15/46) were underestimated, while only 2% (1/46) were equal to, or greater than, the spiked toxin concentration (Table 7 and Table 8). When either spiked or naturally contaminated at, or above, the regulatory limit, the EuroProxima returned 100% (28/28) falsely compliant results (Table 10). A Pearson’s correlation analysis between this rapid kit test and the spiked toxin concentrations was extremely weak at r^2^ = 0.01 (Figure 1). This kit returned a very low mean recovery of 26.7%, well outside the criteria set in the AOAC Guidelines [24] (Table 10).

##### Repeatability of Kits

The repeatability/reliability of all kits was high (standard deviations of the mean ranged from 0.00 to 0.01, with the lower the variation, the higher the reliability of the results). The only exception to this was the Abraxis ELISA kit. From the naturally contaminated Pipi batch with the highest toxin concentration (0.3 OA mg/kg), the repeatability of this kit was low at 0.02 (based on a relatively low number of samples however) (Table 9).

## 3. Discussion

### 3.1. DSTs in Australia

Toxic *Dinophysis* blooms and their impacts remain one of the most problematic HABs worldwide, especially in Mediterranean and European waters [1]. Positive DST detections periodically occur in Australian shellfish, although these events remain largely unstudied [1,27]. Using the official analytical method of LC-MS/MS and LC-MS, shellfish data spanning 2012 to 2017 from four Australian states (Tasmania, Victoria, South Australia and Western Australia) showed that 53 (0.65%) shellfish samples out of the 8156 analyzed exceeded the domestic regulatory limit (0.2 mg OA eq/kg). Exceedances, across all samples combined, for cockles/pipis, clams, mussels, oysters and scallops were 4.9, 1.1, 1.1, 0.03 and 0%, respectively. Of those that exceeded this threshold, OA was the most commonly detected toxin analogue, with only one sample containing DTX-1, and no samples containing DTX-2 (unpublished data).

### 3.2. LC-MS/MS and LC-MS Laboratory Comparison

In the present study, we spiked four different shellfish matrices (SRO, PO, MUS, PIPI) with fixed volumes of relevant CRM to determine the ability of laboratories to quantify DSTs in shellfish using LC-MS/MS and LC-MS. We found that all spiked analogues, OA, DTX-1, and DTX-2, were recovered in all shellfish species across all laboratories, but the results were not consistent across all samples. For example, low and mid-concentration toxin recovery was variable both within and between laboratories (0–150%), while high concentration toxin recovery, which included CRM, was higher, between 60–262%. Two false positives were reported in Pipi samples in which OA was detected at 0.01 and 0.02 mg/kg (Laboratory 1 and Laboratory 2, respectively), and one anomalously high concentration of 2.8 mg/kg was reported from CRM that was submitted at a concentration of 1.07 mg/kg (Table 6). These results need to be interpreted in light of each laboratory’s measurement uncertainty (MU), which was reported as ~10–26%, dependent on the analogue detected (Appendix A). Another issue that must be considered is the homogeneity of toxin within the shellfish, and how that may contribute to the variability in results, particularly at the low- to mid-level spiked concentration.

Finally, we cannot completely discount that there may have been some very low toxin concentrations in these samples which were not detected by the original LC-MS screening. Lab 3, in fact, had the highest level of detection (0.006–0.007 mg/kg for analogues OA, DTX-1 and ±DTX-2) across all the labs used in this study.

In a single laboratory validation study to detect and quantify six lipophilic toxins (azaspiracid, domoic acid, gymnodimine, okadaic acid, pectenotoxin and yessotoxin) in Greenshell mussel, Pacific Oyster, cockle and scallop roe, McNabb et al. [4] reported mean OA recoveries between 92% (from a toxin concentration of 0.5–1.0 mg/kg) and 99% (from a toxin concentration of 0.05–0.10 mg/kg). All six toxins recoveries ranged from 71–99%. As discussed above, this variability was also apparent in our results, albeit in a converse way, whereby shellfish with a higher spiked toxin concentration generally reported a better recovery than those at lower concentrations. McNabb’s study concluded that with some slight methodological adjustments (methanol-water ≅ 9 + 1; 18 mL for 2 g of shellfish tissue), the LC-MS/MS method provides good precision/accuracy and high specificity, and is therefore suitable for the quantification of biotoxins in shellfish for regulatory purposes.

In another study to compare the mouse bioassay (MBA) to electrospray ionization (ESI) LC-MS/MS for the quantification of lipophilic toxins in ~200 samples of shellfish, Suzuki and Quilliam [28] similarly concluded that LC-MS/MS was a powerful tool for both the identification and structure elucidation of many toxins including OA/DTX analogues, but also for the discovery of unknown toxin analogues. Furthermore, studies have shown that LC-MS/MS demonstrates linearity, specificity, repeatability and reproducibility in shellfish samples collected from the environment [29], and is able to resolve the toxin profiles of OA analogues in various *Dinophysis* species isolated from bloom samples [30].

There are, however, disadvantages to using LC-MS/MS and LC-MS for the detection of toxins in shellfish. LC-MS/MS (and LC-MS) is expensive, particularly for farmers in low-risk areas who have a regulatory requirement to undertake marine biotoxin testing using LC-MS/MS at regular intervals (e.g., weekly). The cost is also high for farmers in remote areas, where transport of samples to specialized laboratories is expensive. The LC-MS/MS and LC-MS method is also complex, requiring expert analyst training in dedicated laboratories for sophisticated instrument maintenance and performance. Time delays are another concern, and it can take between 2–7 days to obtain results from a contract laboratory, potentially causing a loss in harvest time and profits to shellfish farmers, and risk to consumers. Finally, high quality and expensive reference material is required to calibrate the method. Despite these disadvantages, and in the absence of a more reliable, sensitive and rapid test, there remains an international acceptance that LC-MS/MS and LC-MS continue to be the standard operating procedure (along with the MBA in many Latin American and Asian countries), for the determination of lipophilic marine biotoxins in mollusks [31].

### 3.3. Rapid Test Kits Comparison

In the search for an inexpensive and reliable alternative method to LC-MS/MS or LC-MS that could be used for screening purposes to serve as an early warning for the shellfish industry, we compared five Rapid test kits against the LC-MS/MS and LC-MS methods. Fifty-five shellfish samples (24 spiked and 30 naturally contaminated pipis) were screened with four quantitative (Beacon, Abraxis and EuroProxima ELISA kits and the Abraxis PP2A kit) and one qualitative (Neogen LFA) rapid test kit to detect OA in Sydney Rock Oysters, Pacific Oysters, Blue Mussels and Pipis. Okadaic acid was the only DST analogue to be tested with these kits for multiple reasons: (i) It has been the dominant analogue detected in Australian shellfish to date; (ii) The cost of purchasing sufficient CRM for spiking all other analogues to detection levels is high; and (iii) Rapid test kit results are reported as µg OA eq/kg, and a spike of varying DST analogues will not reveal individual analogue concentrations (noting the Neogen rapid test kit is qualitative only). Furthermore, each kit reports a level of cross reactivity to the various analogues, and while in most cases this is 100% for OA, it varies for DTX-1 and DTX-2 between kits. For example, if three samples were individually spiked with the same concentration of okadaic acid, DTX-1 and DTX-2, the concentration of okadaic acid from the Abraxis ELISA kit would read as double the concentrations of the other two compounds. This is because DTX-1 (50%) and DTX-2 (50%) only give half of the response that okadaic acid does with this technology.

With this in mind, all quantitative kits should theoretically provide a comparable concentration of OA to that obtained using the LC-MS method. Regression analyses showed the correlations between the ELISA Rapid test kits and LC-MS in our study were all very low (0.002–0.19), while the correlation between the PP2A Abraxis kit and LC-MS was moderate to high (0.72) (Figure 1). The observed variations between these methods could not be attributed to matrix effects however, as no clear differences were observed between spiked samples across methods. Certain kits nonetheless performed better on naturally contaminated samples (Pipis only) compared to spiked samples (Neogen and Abraxis PP2A). The reasons for this remain unclear, but support the assertion by Turner et al. [32] that validation studies need to include both relevant shellfish species and naturally contaminated shellfish samples, so that any rapid test kit performance is measured using local toxin profiles.

After the development of the first ELISA method by Dubois et al. [33], a comparison across assay techniques was undertaken whereby cell counts, LC-MS/MS, the newly developed Abraxis ELISA and PP2A Okatests were compared. Naturally contaminated samples of edible Blue Mussels (*Mytilus edulis*) were examined for total DST toxin content including esters and DTX-3. The ELISA showed matrix effects on hydrolyzed samples, which had both high and low levels of toxins, while the PP2A adequately detected both low and high DST concentrations in mussel samples. While the Okatest was recommended in preference to the ELISA, it was concluded to be a specific assay (could not detect other regulated DSTs), and therefore could not replace LC-MS/MS or LC-MS. Subsequent to these findings, three further studies—a single laboratory validation and an interlaboratory study on the PP2A Okatest [34,35], and a comparison across three RTKs (the lateral flow (Jellett/Scotia), ELISA (Abraxis) and PPIA (Okatest) kits) [26] were undertaken. Considering issues such as an unacceptable number of false negatives (Jellett), and low cross-reactivity with DTX-1 (the dominant toxin profile in the shellfish tested) by the ELISA, Eberhart et al. concluded that the PP2A was the most promising kit on the market. It is these differences in toxin profiles, the inclusion (or not) of a hydrolysis step, and whether the shellfish tested is spiked or naturally contaminated, that prevents a direct comparison between these studies and the present study, although it highlights the issues that must be standardized in any future validation study. 

In 2015, Jawaid et al. reported on the development and validation of a new rapid test kit, the Neogen LFA, this time a qualitative test strip/reader for the OA group toxins in shellfish [19]. This validation method tested both spiked (OA, DTX-1, DTX-2 and DTX-3 with hydrolysis procedure) and naturally contaminated shellfish (mussels, scallops, oysters, and clams) and compared the results to LC-MS/MS. While our study showed only minor differences in shellfish matrices (low number of samples tested however) and zero falsely compliant results in naturally contaminated samples, Jawaid et al. showed no matrix effects, false compliant results or false noncompliant results at <50% MPL (maximum permitted level). Both Jawaid and the present study suggest that this method, with some further work, may be an effective early warning tool for the shellfish industry. The results reported in this study, however, do not support the use of any DST rapid test kit as a stand-alone quality assurance measure at this time, and further research and development work is needed.

Since the development of the LFA technology, two additional studies generated rapid test kit comparisons [25,36]. The first study compared DSTs in shellfish from Argentina using two qualitative lateral flow kits (Scotia and Neogen), the quantitative PPIA kit (OkaTest), and the ELISA kit (Max Signal—no longer commercially available) and compared the results to LC-MS/MS. The specificity was reported as good for all kits, with no false compliant results against the ML of <0.16 mg OA eq/kg). The second study screened four RTKs, again on naturally contaminated shellfish, but this time from Great Britain. The quantitative PP2A (OkaTest) was the only test to show the complete absence of falsely compliant results (i.e., mussel samples containing OA-group toxins above the MPL of 0.16 mg OA eq/kg which returned negative results) and showed a fair correlation to LC-MS/MS but with an overall overestimation of sample toxicity with some indication of matrix effect, particularly in oysters [36]. The quantitative ELISA (MaxSignal) gave a reasonable correlation with LC-MS/MS, no evidence of overestimation, accuracy at low concentrations and only one falsely compliant result (as above, a mussel samples containing OA-group toxins above the MPL of 0.16 mg OA eq/kg which returned a negative result). The two lateral flow assays (Neogen and Scotia) were observed to show high agreement with LC-MS/MS and no indications of false positives), although both returned one false negative [36].

In the present study, all four quantitative kits showed varying levels of over/underestimation (many at the regulatory limit). Many results were outside the working range or limit of these kits. This ranged from 29% of samples using the Abraxis PP2A to 65% with the EuroProxima ELISA (Table 10). Two kits also showed false positives from blank matrices (i.e., samples that did not contain toxins), these being the Abraxis PP2A and EuroProxima ELISA at 25% and 13% respectively. We cannot, however, discount the fact that there may have been some very low toxin concentrations in these samples which were not detected by LC-MS. All methods (quantitative and qualitative) delivered high levels (25% to 100%) of falsely compliant results for spiked samples. The Neogen and Abraxis PP2A performed satisfactorily (0%, 5% falsely compliant at the regulatory limit or above, respectively) for naturally contaminated pipis. The mean percent recovery ranged from 27% (EuroProxima ELISA) to 107% (LC-MS), while only the LC-MS method and the Abraxis PP2A kit (92%) fell within the “acceptable recovery” range of 80–100% as set by the AOAC Guidelines [24].

## 4. Conclusions

Overall, considering the highly varied, and sometimes erroneous results, along with other factors such as method cost, preparation time, test complexity, and extra equipment required, the disadvantages of using the currently available rapid test kits are considerable (Table 9. Quantitatively, the Abraxis PP2A kit outperformed all other rapid test kits (notably in naturally contaminated pipis)) and may be suitable for screening purposes. In using this kit, however, one sample took ~3 h to complete. This kit also requires more rigorous testing to determine the statistics around its false compliant results. Continued collaboration with the manufacturer to refine this test procedure should be undertaken to improve its potential. Qualitatively, the Neogen test kit performed well for naturally contaminated Pipis (0% falsely compliant results at the regulatory level) but appeared much less reliable (63% false negative results at regulatory level) for spiked pipis, oysters, and mussels. These results suggest possible differences in kit performance dependent on the shellfish matrix analyzed, or whether the shellfish is naturally contaminated or artificially spiked. The reason(s) for differing results between naturally contaminated shellfish and spiked samples, however, remains unclear, particularly when toxin determination using LC-MS did not result in any significant difference between these two routes in the present study. The Neogen kit is, however, relatively simple to use, returns a faster result than other kits, and, as discussed above, shows promising results for naturally contaminated shellfish. A single laboratory validation study, such as the one carried out by for paralytic shellfish toxins in mussels and oysters [37], followed by an international validation study, is recommended prior to approval of any rapid test kit for regulatory purposes.

## 5. Materials and Methods

### 5.1. Interlaboratory Comparison for LC-MS/MS and LC-MS

#### 5.1.1. Shellfish Preparation

Sample preparation was based on the standard operating procedure for the determination of lipophilic marine biotoxins in molluscs by LC-MS/MS and LC-MS. Specifically, raw samples of Sydney Rock Oysters (*Saccostrea glomerata*), Pacific Oysters (*Magallana gigas*/*Crassostrea gigas*), Blue Mussels (*Mytilus galloprovincialis*) and Pipis (*Plebidonax deltoides*/*Donax deltoides*) were sourced from the Sydney Fish Markets on 6/6/2019. From here on, these matrices are referred to as SRO, PO, MUS and PIPI, respectively. These were stored at 4–8 °C and transported immediately to the laboratory for processing. All shellfish were washed thoroughly with fresh water, shucked (if necessary) and tissue was removed. Stock material of each species was made by pooling the tissue of 3–6 individuals (for each spike treatment) of that species, homogenizing and spiking with fixed volumes of relevant standards (see below) and homogenizing again. Subsamples of this species-specific tissue homogenate were then accurately weighed (~3 g) and aliquoted into 5 mL polypropylene Bacto sample jars (Model No. SCP5014UU) and frozen at −20 °C until they were dispatched to contract laboratories for toxin determination by LC-MS/MS and LC-MS.

#### 5.1.2. Standard Reference Materials

Certified reference materials (CRMs) were purchased from the National Research Council Canada (NRC) for shellfish spiking and quality control testing. These included: (i) CRM DSP-Mus-c which is a thermally sterilized homogenate (4.0 ± 0.75 g) of mussel tissue (*Mytilus edulis*) and the dinoflagellate *Prorocentrum lima*, with toxin levels of okadaic acid (OA), dinophysistoxin-1 (DTX-1) and dinophysistoxin-2 (DTX-2) at 1.07 ± 0.08 µg/g, 1.07 ± 0.11 µg/g and 0.86 ± 0.08 µg/g, respectively (positive control); (ii) CRM-OA-d which contained ~0.5 mL of a solution of OA in methanol at a concentration of 8.4 ± 0.4 µg/mL; (iii) CRM-DTX-1-b which contained ~0.5 mL of a solution of dinophysistoxin 1 (DTX-1) in methanol at a concentration of 7.8 ± 0.5 µg/mL; and (iv) CRM-DTX-2-b which contained ~0.5 mL of a solution of dinophysistoxin-2 (DTX-2) in methanol at a concentration of 3.8 ± 0.2 µg/mL.

#### 5.1.3. Spiking of Shellfish Matrices

A subsample (3 g) of each pooled, species-specific matrix (SRO, PO, MUS and PIPI) was first analyzed by LC-MS at Laboratory 3 (see below) to ensure each matrix contained no DSTs before the experiment began (limit of detection (LOD) = 0.006–0.007 mg/kg for analogues OA, DTX-1 and ± DTX-2) (Appendix A).

Spiking of each species-specific homogenate with a range of DST concentrations then followed for both LC-MS/MS and LC-MS. These concentrations were chosen based on the capability of most laboratories to achieve a limit of reporting (LOR) of ~0.01 mg/kg (Table 11, Appendix A). In brief, one batch of each matrix was spiked with OA (≅7.2 µL/3g, which is equivalent to 2 × LOR (0.02 mg/kg); the second one with DTX-1(≅14.0 µL/3g, which is 4 × LOR (0.04 mg/kg), and the third with DTX-2 (≅8 µL/3g, which is equivalent to the LOR (0.01 mg/kg). While increasing the spiking concentration of this latter analogue would provide a more rigorous comparison of the laboratories capabilities, our decision to spike DTX-2 at the LOR was based on cost and the infrequency of this analogue identified in Australian shellfish to date. A ~3 g aliquot of each of these species-specific homogenates was then sent to each laboratory to test their LOR and any matrix effect (Table 11).

Next, a second species-specific homogenate was spiked with a combination of all three toxins: 35 µL/3 g OA for SRO and PO which is 10 × LOR (0.1 mg/kg) or 7.2 µL/3g OA for MUS and PIPI which is equivalent to 2 × LOR (0.02 mg/kg); 17.6 µL/3 g DTX-1 which is 5 × LOR (0.05 mg/kg) into all shellfish species; and 16 µg/3 g DTX-2 which is 2 × LOR (0.02 mg/kg) again into all shellfish species. These combination-spiked samples were then aliquoted (~3 g) and sent to each laboratory to test toxin profile detection capability and also any matrix effect (Table 11).

Furthermore, to test the reproducibility/repeatability of each laboratory, a third batch of the SRO homogenate was spiked with OA (≅7.2 µL/3 g which is equivalent to 2 × LOR (0.02 mg/kg) and three replicate aliquots of this stock material (3 g) were dispatched to each laboratory. Finally, one sample (~3 g) of the CRM DSP-Mus-c was sent to each laboratory as a positive control. In total, 19 samples (randomly numbered 1–19) were dispatched frozen to each of four laboratories (Table 11).

#### 5.1.4. LC-MS/MS and LC-MS Toxin Determination

Four commercial and/or government analytical laboratories with experience in conducting LC-MS/MS and LC-MS of marine biotoxins in shellfish were engaged to determine DSTs in spiked shellfish, identified only as Laboratories 1–4. The aim of this part of the study was to determine an inter-laboratory comparison of standardized samples, in order to obtain a baseline result using currently mandated seafood safety procedures in Australia [38]. The LC-MS/MS and LC-MS methods were engaged by each of the laboratories, and their limits of detection and limits of reporting/quantification are shown in Appendix A. No recovery corrections were applied to the final results reported from any of the labs.

### 5.2. Rapid Test Kit Comparison

#### 5.2.1. Shellfish Preparation

Raw samples of SRO, PO, MUS and PIPI (same species as above), were sourced from the Sydney Fish Markets on 29/4/2020. These were stored at 4–8 °C and transported immediately to the University of Technology Sydney laboratory for processing. Again, all shellfish were washed thoroughly with fresh water, shucked and tissue was removed. Bulk material of each species was then made by pooling the tissue of individuals of that species up to 90 g, homogenizing and separating into 3 batches for downstream processing. The first batch served as unspiked controls and were first examined by LC-MS at Laboratory 3 (see above) to ensure each matrix was clear of toxins before the experiment began. The second batch was spiked with CRM-OA-d at ~12 µL/g (0.1 OA eq. mg/kg), which is half the regulatory limit, and the third batch was spiked at ~24 µL/g, which is equal to the regulatory limit. Once prepared all batches were returned to the freezer (−20 °C) until further processing.

Additionally, during Oct/Nov 2019, DSTs were detected in wild harvest Pipis from Sydney Fish Markets (~400 mg/kg), and a recall was immediately actioned. A batch of these naturally contaminated Pipis were obtained and prepared as positive controls: Sample 4A—14/11/19 Stockton 4–6 km; 4B—7/11/19 Stockton 4 km; 4C—31/10/19 Stockton 2–4 km; and 4D—Sydney Fish Market Stockton recall Nov 2019. Once the OA toxin concentration was determined using LC-MS for these environmentally contaminated samples, samples with toxin level closest to the regulatory level (0.2 mg OA eq/kg) were chosen, and 10 replicates of these positive controls were run on each kit to test the reliability/repeatability of each kit.

A subsample (3 g) of each pooled, species-specific matrix was first examined by LC-MS (Laboratory 3) to ensure each matrix was clear of toxins before the experiment began (unspiked controls). All remaining batches (spiked and positive controls) were then subsampled and prepared according to the rapid test kit protocols for each kit or for LC-MS analysis. Duplicate samples of each treatment/shellfish were tested using both LC-MS and the five test kits.

#### 5.2.2. Rapid Test Kits

A list of DST rapid test kits were screened, their method details including their limit of quantification or working range, amount of tissue required, cost, time for analysis etc., are summarized in Table 9.

##### Qualitative Test

##### Neogen 

Neogen Reveal 2.1 DSP Test strips (Lot: 9561-49, Neogen Corporation, Scotland, UK) and DSP hydrolysis packs (Lot: 9555-09) were stored at room temperature until experiments began. Each shellfish sample (2 g) was defrosted to room temperature (20–25 °C), then transferred to the extraction bag provided before being homogenized with 8 mL analytical grade methanol (Sigma-Aldrich, Sydney, Australia). The sample extract was then poured from each extraction bag (from opposite side of mesh divider) into a 15 mL falcon tube, prior to filtration using a 0.45 µm sterile Minisart^®^ syringe filter into another clean 15 mL tube. Eighty µL of filtered extract was then transferred to a clean glass vial, followed by 100 µL of 2.5 M NaOH, before being capped tightly and mixed using a vortex on full speed for 30 s. The sample vial was then transferred to a heater block set at 76 °C for 40 min, after which time the sample was cooled on ice. At room temperature, 100 µL of 2.5 M HCl was added to the sample extract, mixed by hand for 30 s, before 100 µL transferred into a DSP buffer A vial (provided). The sample was again vigorously mixed, before 100 µL was transferred to a microwell plate. A DSP strip was then placed into the microwell plate for 15 min before being immediately placed into the AccuScan^®^ PRO 2.0 scanner for result interpretation.

##### Quantitative Tests

##### Abraxis PP2A

The Eurofins/Abraxis Okadaic Acid (PP2A) Microtiter Plate kit Product No. 520025, Lot No. 19/1259, Eurofins Abraxis, Warminster, PA, USA) was stored at 4 °C prior to use. Upon opening, the solutions were prepared as per the manufacturer’s protocols and allowed to reach room temperature before analysis began. Each shellfish subsample (5 g) was defrosted and 25 mL methanol (Sigma-Aldrich, Sydney, Australia) added before homogenization in a tube shaker for 2 min. The sample was then centrifuged at 2000× *g* for 10 min at 4 °C and 640 µL of the methanolic extract removed and transferred to a clean 15 mL falcon tube. The extract was then mixed with 100 µL of 2.5 N NaOH, sealed, and placed in a water bath at 76 ± 2 °C for 40 min. After removal from the water bath, 80 µL of 2.5 N HCl was added to each sample, followed by 20 mL buffer solution.

For the test protocol, a volume of 50 µL of each OA standard (provided at 0.5, 0.8, 1.2, 1.8, and 2.8 nM) and each shellfish sample was added to the 96 well-plate provided. To each of these wells, 70 µL phosphatase solution was added. The plate was then tapped gently to ensure mixing, before being covered with parafilm and incubated for 20 min at 30 ± 2 °C. Immediately after this incubation period, 90 µL of chromogenic substrate was added to each well, and again, the plate was tapped gently to ensure mixing. The plate was then incubated (covered) for a further 30 min at 30 °C ± 2 °C, after which 70 µL of stop solution was added to each well. Absorbance was immediately read at 405 nm using a Tecan Infinite M1000 PRO plate reader.

For data analysis, a standard curve was obtained by plotting the absorbance values in a linear *y*-axis and the concentration of okadaic acid in a logarithmic *x*-axis. The OA concentration contained in the sample (Cs) was then calculated using the following equation:

x = EXP ((y − b)/a),(1)

where x was the OA concentration in the sample (Cs) and y the absorbance of the sample. The concentration of DSTs in tissue (Ct) was then determined as:

Ct (mg/kg) = ((Cs (nM) × FD × MW (g/mol) × Ve (L))/Mt (g))/1000(2)

where Ct: DST concentration in tissue expressed as equivalents of OA; Cs: toxins concentration in sample; FD: Methanolic extract dilution factor (i.e., 640 µL/20 mL → × 31.25); MW: Okadaic acid molecular weight = 805; Ve: Methanolic extract volume (0.025 L); Mt: Tissue weight (5 g).

##### Beacon ELISA

The Beacon Okadaic Acid (ELISA) Plate kit (Cat. No. 20-0184, Lot No. 6289J, Beacon Analytical Systems Inc., Sako, ME, USA) was stored at 4 °C and all reagents brought to room temperature before use. Each shellfish sample (1 g) was defrosted and 2 mL 80% methanol (Sigma-Aldrich, Sydney, Australia)/water was added before homogenization and transfer to a clean 15 mL falcon tube. A further 8 mL of 80% methanol/water was then added, before vortexing for 5 min followed by centrifugation at 3000 rpm for 5 min. The supernatant was then filtered into a clean 15 mL tube through a 0.45 µm sterile Minisart^®^ syringe filter and the extract diluted 1:50 into 10% methanol/10 mM PBS (Sigma-Aldrich, Sydney, Australia) (i.e., 40 µL of filtered extract into 1.96 mL of 10% methanol/10 mM PBS).

For the test procedure, 50 µL of enzyme conjugate was added into each test well, followed by 100 µL of each OA calibrator (provided at 0, 0.2, 0.5,1.2 and 5 µg/L) or shellfish sample, and 50 µL of antibody. Wells were then mixed for 30 s using gentle shaking, followed by incubation at room temperature for 30 min. The content of the well plates were then decanted, and well plates were washed four times using Milli-Q water, and inverting the plate onto absorbent paper between each wash. After the final wash, 100 µL of substrate was added to each well, before incubation for 30 min at room temperature. Finally, 100 µL of stop solution was added to each well and absorbance read at 450 nm using the Tecan Infinite M1000 PRO plate reader.

For quantitative interpretation of the absorbance readings, a standard curve was then constructed by plotting the absorbance of the calibrators (standards) on the *y*-axis versus the concentration of okadaic acid in a logarithmic *x*-axis. The OA concentration (ppb) contained in the sample (Cs) was then calculated using Equation (1) above. Finally, to obtain the final DST (mg/kg) in each sample, a factor of ×500 to account for the dilution during the shellfish extraction step was applied.

##### Abraxis ELISA

The Eurofins/Abraxis Okadaic Acid (DSP) ELISA, Microtiter Plate (Product No. 520021, Lot No. 19/1178, Eurofins Abraxis, Warminster, PA, USA) was stored at 4 °C and brought to room temperature before use. All solutions were prepared as per the manufacturer’s protocols. Each shellfish subsample (1 g) was defrosted and 6 mL methanol (Sigma-Aldrich, Sydney, Australia)/Milli-Q water (80/20) added before homogenization for 2 min. Each sample was then centrifuged for 10 min at 3000× *g* and the supernatant was transferred to a clean 15 mL falcon tube. A further 2 mL methanol/Milli-Q was added to the shellfish residue, the sample centrifuged again for 10 min at 3000× *g*, and the supernatant added to the first portion. The final volume was brought up to 10 mL with methanol/Milli-Q, before filtration into a clean 15 mL tube through a 0.45 µm sterile Minisart^®^ syringe filter. For the hydrolysis step, 500 µL of each sample extract was added to a 2 mL glass vial, and 100 µL of 1.25 N NaOH added. The sample was then vortexed for 15–20 s before incubation on a heat block at 80 °C for 40 min. Each sample was then cooled and 100 µL of 1.25 N HCl added and vortexed for 15–20 s. Finally, 10 µL of the hydrolyzed extract was mixed with 990 µL of 1× sample diluent (1:100 dilution) in a 2 mL glass vial with a cap and vortexed again.

For the assay procedure, a volume of 100 µL of each OA standard (provided at 0, 0.1, 0.2, 0.5, 1, 2, 5 ppb) and shellfish sample was added to each strip well and placed into the well plate provided. To each of these, 50 µL of enzyme conjugate and 50 µL of antibody solution was added. The plate was then covered with parafilm, rotated carefully to mix and left to incubate for 60 min at room temperature, after which the covering was removed and the contents were decanted by inverting the plate onto a paper towel. Each well was then thoroughly washed three times using the diluted wash buffer (~25 µL for each wash/each well), blotting after each step. Following the final washing step, 150 µL of substrate solution was added to each well, before covering with parafilm, rotating gently to mix, and incubating at room temperature for 30 min. Finally, 100 µL of stop solution was added to each well plate prior to immediate absorbance reading at 450 nm using the Tecan Infinite M1000 PRO plate reader.

Kit performance was evaluated by calculating %B/Bo for each standard by dividing the absorbance value for each standard by the Zero standard mean absorbance. A standard curve was then constructed by plotting the %B/Bo for each standard on the *y*-axis versus the concentration of okadaic acid in a logarithmic *x*-axis. The OA concentration (ppb) contained in the sample (Cs) was then calculated using Equation (1) above. Finally, to account for hydrolysis sample extraction, hydrolysis and dilutions during the hydrolysis step, all results were multiplied × 1400 to obtain the DSP concentration (ppb) before conversion to mg/kg.

##### EuroProxima ELISA

The EuroProxima Okadaic Acid ELISA (Catalogue No. 5191OKA, Lot No. UN6635, Arnhem, The Netherlands) was stored at 4 °C before use and subsequently brought to room temperature before use. Reagents were prepared as specified in the manufacturer’s protocol. To begin, 1 mL of water was added to each 1 g of shellfish, the sample vortexed for 1 min, and a further 2 mL of 100% methanol (Sigma-Aldrich, Sydney, Australia) was added. The sample was again vortexed for 1 min followed by centrifugation at 2000× *g* for 10 min. The clear supernatant was then filtered using a 0.45 µm sterile Minisart^®^ syringe filter into a clean 15 mL falcon tube and the sample subsequently diluted 1:50 with the sample dilution buffer provided.

For the assay procedure, 100 µL of the zero standard (0 ng/mL) was pipetted into the first well, and 50 µL thereafter of each OA standard (provided at 0, 0.2, 0.5, 1.0, 2.0, 5.0 10.0 ng/mL) and shellfish samples into the 96 well-plate provided. Following on, 25 µL of enzyme conjugate and 25 µL of antibody was added to each well, except A1. The plate was then sealed with parafilm and gently shaken for 1 min before incubation at room temperature for 30 min. Parafilm was subsequently removed, the well contents discarded onto absorbent paper, and all wells were washed three times with a rinsing buffer. After the final rinse, 100 µL of substrate solution was added to each well, mixed thoroughly and left to incubate for 15 min in the dark prior to 100 mL of stop solution being added. Absorbance was read at 450 nm using the Tecan Infinite M1000 PRO plate reader.

For data interpretation, the mean optical density (OD) value of the wells A1 and A2 were subtracted from the individual OD reading from each of the standards and samples. The OD values of the six standards and samples are then divided by the OD value of the zero standard (well no. B1) and multiplied by 100. The zero standard is then equal to 100% (maximum OD) and the other OD values are % of the maximal OD. A calibration curve was then constructed with the values (% maximal OD) plotted on the y-axis versus the concentration of okadaic acid (ng/mL) in a logarithmic *x*-axis. The OA concentration (ng/mL) contained in the sample (Cs) was then calculated using Equation (1) above, but this time where x was the OA concentration in the sample (Cs) and y the % max OD of the sample. Finally, to obtain OA equivalents in the final shellfish, a factor of × 200 (and/1000) was applied.

### 5.3. Data Assessment

Toxin recovery from samples analyzed using LC-MS/MS and LC-MS were assessed in four ways. 1. Where sample replication was available, mean (±SD) toxin recoveries were calculated and compared to the spiked concentration and LOR, and finally compared across laboratories. 2. To determine each analogue recovery using LC-MS/MS and LC-MS, toxin results from each shellfish species were compared to the spiked toxin concentration, and then compared across laboratories. 3. For shellfish that were spiked with a combination of OA analogues, the results were compared to both spiked concentration and the ML (0.2 mg/kg OA), as well as across laboratories. 4. Finally, the recovery of toxins in certified reference material CRM (DSP-Mus-c) were compared across laboratories.

To examine the performance of the rapid test kits, firstly, we assessed the performance of the qualitative Neogen kit by comparison to the spiked toxin concentration in each sample (% false positives/% false negatives). Secondly, the performance and recovery of all quantitative methods (including LC-MS) were compared (% overestimated; % underestimated; % recovery; Pearson’s correlation using Excel 2016) to the spiked concentration of each sample. For those samples spiked at, or above, the ML adopted by the Food Standards Australia New Zealand (0.2 OA mg/kg), we also determined whether they were “falsely compliant” or “falsely non-complaint” with the ML. These terms refer to the comparison of the results obtained to the maximum regulatory limit. For example, if a sample was spiked above the regulatory limit but resulted in a concentration below the regulatory limit, it was referred to as “falsely compliant”. Conversely, if a sample was spiked below the regulatory limit but returned a concentration above the regulatory limit, it was referred to as “falsely non-compliant”. Thirdly, a comparison across species-specific matrices was undertaken to assess the suitability of rapid test kits across a range of shellfish species. Finally, the reliability or repeatability of each kit was assessed (defined as the standard deviation of the mean, Excel 2016) from the replicate positive controls (naturally contaminated Pipi samples) across all quantitative kits.

## Figures and Tables

**Table 1:** Results of LC-MS/MS (Liquid Chromatography—tandem Mass Spectrometry) and LC-MS (Liquid Chroma-tography—Mass Spectrometry) for Sydney Rock Oysters (SRO) spiked with 0.02 mg/kg okadaic acid (no DTX-1 or DTX-2 added).

Replicate	Species	Analyte	Spike	Lab 1	Lab 2	Lab 3	Lab 4
	Code	Code	mg/kg	mg/kg	mg/kg	mg/kg	mg/kg
1	SRO	OA Free	0.02	0.01	<0.01	0.01	<0.025
	SRO	OA Total	0.02	0.01	<0.01	0.01	<0.025
2	SRO	OA Free	0.02	0.02	<0.01	0.01	<0.025
	SRO	OA Total	0.02	<0.01	<0.01	0.01	<0.025
3	SRO	OA Free	0.02	0.01	<0.01	0.02	<0.025
	SRO	OA Total	0.02	0.01	<0.01	0.02	<0.025

<LOR = below limit of reporting; Note: Spike below limit of reporting for Laboratory 4.

**Table 2:** Results of LC-MS/MS and LC-MS for Australian shellfish—Sydney Rock Oysters (SRO), Pacific Oysters (PO), Blue Mussels (MUS) and Pipis (PIPI) spiked with 0.02 mg/kg okadaic acid (no DTX-1 or DTX-2 added).

Sample	Species	Analyte	Spike	Lab 1	Lab 2	Lab 3	Lab 4
	Code	Code	mg/kg	mg/kg	mg/kg	mg/kg	mg/kg
1	SRO	OA Free	0.02	0.01	<0.01	0.02	<0.025
	SRO	OA Total	0.02	0.01	<0.01	0.02	<0.025
2	PO	OA Free	0.02	0.02	<0.01	<0.01	<0.025
	PO	OA Total	0.02	0.02	<0.01	<0.01	<0.025
3	MUS	OA Free	0.02	0.02	0.01	<0.01	<0.025
	MUS	OA Total	0.02	0.01	0.01	<0.01	<0.025
4	PIPI	OA Free	0.02	0.01	<0.01	0.01	<0.025
	PIPI	OA Total	0.02	0.01	0.02	0.01	0.03

<LOR = below limit of reporting; Note: Spike below limit of reporting for Laboratory 4.

**Table 3:** Results of LC-MS/MS and LC-MS for Australian shellfish—Sydney Rock Oysters (SRO), Pacific Oysters (PO), Blue Mussels (MUS) and Pipis (PIPI) spiked with 0.04 mg/kg DTX-1 (no OA or DTX-2 added).

Sample	Species	Analyte	Spike	Lab 1	Lab 2	Lab 3	Lab 4
	Code	Code	mg/kg	mg/kg	mg/kg	mg/kg	mg/kg
1	SRO	DTX-1 Free	0.04	0.05	0.02	0.03	0.04
	SRO	DTX-1 Total	0.04	0.04	0.02	0.03	0.026
2	PO	DTX-1 Free	0.04	0.04	0.02	0.02	0.03
	PO	DTX-1 Total	0.04	0.04	0.02	0.02	<0.025
3	MUS	DTX-1 Free	0.04	0.04	0.03	0.02	<0.025
	MUS	DTX-1 Total	0.04	0.03	0.03	0.02	<0.025
4	PIPI	DTX-1 Free	0.04	0.05	0.02	0.03	0.031
	PIPI	DTX-1 Total	0.04	0.03	0.03	0.03	<0.025
	PIPI	OA Total	-	0.01	0.02	<0.01	<0.025

<LOR = below limit of reporting.

**Table 4:** Results of LC-MS/MS and LC-MS for Australian shellfish—Sydney Rock Oysters (SRO), Pacific Oysters (PO), Blue Mussels (MUS) and Pipis (PIPI) spiked with 0.01 mg/kg DTX-2 (no OA or DTX-1 added).

Sample	Species	Analyte	Spike	Lab 1	Lab 2	Lab 3	Lab 4
	Code	Code	mg/kg	mg/kg	mg/kg	mg/kg	mg/kg
1	SRO	DTX-2 Free	0.01	<0.01	<0.01	<0.01	<0.015
	SRO	DTX-2 Total	0.01	<0.01	<0.01	<0.01	<0.015
2	PO	DTX-2 Free	0.01	0.01	<0.01	<0.01	<0.015
	PO	DTX-2 Total	0.01	<0.01	<0.01	<0.01	<0.015
3	MUS	DTX-2 Free	0.01	0.01	<0.01	<0.01	<0.015
	MUS	DTX-2 Total	0.01	<0.01	<0.01	<0.01	<0.015
4	PIPI	DTX-2 Free	0.01	<0.01	<0.01	<0.01	<0.015
	PIPI	DTX-2 Total	0.01	<0.01	<0.01	<0.01	<0.015

<LOR = below limit of reporting; Note: Spike below limit of reporting for Laboratory 4.

**Table 5:** Results of LC-MS/MS and LC-MS for Australian shellfish—Sydney Rock Oysters (SRO), Pacific Oysters (PO), Blue Mussels (MUS) and Pipis (PIPI) spiked with a combination of DST analogues-OA 0.1 mg/kg; DTX-1 0.05 mg/kg; and DTX-2 0.02 mg/kg.

Sample	Species	Analyte	Spike	Lab 1	Lab 2	Lab 3	Lab 4
	Code	Code	mg/kg	mg/kg	mg/kg	mg/kg	mg/kg
1	SRO	DTX-1 Free	0.05	0.05	0.01	0.02	0.038
	SRO	DTX-1 Total	0.05	0.04	0.02	0.02	0.03
	SRO	DTX-2 Free	0.02	0.02	<0.01	0.01	<0.015
	SRO	DTX-2 Total	0.02	0.01	<0.01	0.01	<0.015
	SRO	OA Free	0.1	0.05	0.04	0.04	0.089
	SRO	OA Total	0.1	0.04	0.04	0.04	0.062
2	PO	DTX-1 Free	0.05	0.03	0.02	0.02	0.036
	PO	DTX-1 Total	0.05	0.03	0.03	0.02	0.029
	PO	DTX-2 Free	0.02	0.02	<0.01	0.01	<0.015
	PO	DTX-2 Total	0.02	0.02	<0.01	0.01	<0.015
	PO	OA Free	0.1	0.06	0.04	0.04	0.08
	PO	OA Total	0.1	0.04	0.05	0.04	0.067
3	MUS	DTX-1 Free	0.05	0.02	0.03	0.02	0.03
	MUS	DTX-1 Total	0.05	0.03	0.03	0.02	<0.025
	MUS	DTX-2 Free	0.02	0.01	0.01	0.01	<0.015
	MUS	DTX-2 Total	0.02	0.02	0.01	0.01	<0.015
	MUS	OA Free	0.01	0.01	0.01	<0.01	<0.025
	MUS	OA Total	0.01	0.01	<0.01	<0.01	<0.025
4	PIPI	DTX-1 Free	0.05	0.03	0.03	0.01	0.033
	PIPI	DTX-1 Total	0.05	0.03	0.03	0.01	0.036
	PIPI	DTX-2 Free	0.02	0.02	0.02	<0.01	<0.015
	PIPI	DTX-2 Total	0.02	0.01	<0.01	<0.01	<0.015
	PIPI	OA Free	0.01	0.02	0.01	<0.01	<0.025
	PIPI	OA Total	0.01	0.02	0.04	<0.01	0.034

<LOR = below limit of reporting; Note: Spike of OA for MUS and PIPI below limit of reporting for Laboratory 4.

**Table 6:** Results of LC-MS/MS and LC-MS for Certified Reference Material CRM DSP-Mus-c.

Sample	Species	Analyte	Concentration	Lab 1	Lab 2	Lab 3	Lab 4
	Code	Code	mg/kg	mg/kg	mg/kg	mg/kg	mg/kg
1	+CONT	DTX-1 Free	1.07	1.4	0.87	0.91	1.1
	+CONT	DTX-1 Total	1.1 *	1.4	1.04	2.31	1.3
	+CONT	DTX-2 Free	0.86	0.76	0.68	0.82	0.87
	+CONT	DTX-2 Total	2.2 *	2.0	1.97	1.32	2.6
	+CONT	OA Free	1.07	1.1	0.85	0.89	2.8
	+CONT	OA Total	2.4 *	2.2	2.29	1.79	5.0

* CRM are certified for free toxin; they report higher total toxin concentration post hydrolysis but these are not certified.

**Table 7:** Results of LC-MS and rapid test kits for Okadaic Acid spiked into Australian shellfish (Sydney Rock Oysters [SRO], Pacific Oyster [PO], Blue Mussel [MUS] and Pipis [PIPI]). Note: Neogen qualitative test (±) with Limit of Quantification = 0.08 mg/kg; Abraxis PP2A Working Range = 0.06 to 0.35 mg/kg; Beacon ELISA Limit of Quantification = 0.1 mg/kg; Abraxis ELISA Working Range = 0.1–5.0 mg/kg; Europroxima ELISA Limit of Quantification = 0.04 mg/kg.

Sample No. and Shellfish Matrix	OA mg/kg	LC-MS	Neogen	Abraxis PP2A	Beacon ELISA	Abraxis ELISA	Europroxima ELISA
Sample 1 (SRO)	-	ND	-	0.02	0.05	0.00	0.03
Sample 2 (SRO)	-	ND	-	0.07	0.06	0.03	0.01
Sample 3 (SRO)	0.1	0.12	-	0.05	0.12	0.00	0.04
Sample 4 (SRO)	0.1	0.13	-	0.02	0.11	0.01	0.19
Sample 5 (SRO)	0.2	0.23	+	0.17	0.18	0.01	0.08
Sample 6 (SRO)	0.2	0.23	-	0.05	0.24	0.01	0.09
Sample 7 (PO)	-	ND	-	0.07	0.07	0.00	0.08
Sample 8 (PO)	-	ND	-	0.03	0.05	0.00	0.02
Sample 9 (PO)	0.1	0.12	-	0.05	0.12	0.01	0.04
Sample 10 (PO)	0.1	0.17	-	0.11	0.18	0.03	0.04
Sample 11 (PO)	0.2	0.23	-	0.12	0.20	0.03	0.04
Sample 12 (PO)	0.2	0.23	-	0.21	0.20	0.03	0.07
Sample 13 (MUS)	-	ND	-	0.02	0.05	0.03	0.01
Sample 14 (MUS)	-	ND	-	0.03	0.06	0.02	0.02
Sample 15 (MUS)	0.1	0.19	-	0.16	0.12	0.01	0.09
Sample 16 (MUS)	0.1	0.17	-	0.06	0.10	0.01	0.02
Sample 17 (MUS)	0.2	0.23	+	0.16	0.21	0.01	0.11
Sample 18 (MUS)	0.2	0.23	-	0.08	0.19	0.02	0.04
Sample 19 (PIPI)	-	ND	-	0.04	0.09	0.01	0.02
Sample 20 (PIPI)	-	ND	-	0.02	0.09	0.01	0.01
Sample 21 (PIPI)	0.1	0.1	-	0.13	0.17	0.02	0.04
Sample 22 (PIPI)	0.1	0.09	-	0.05	0.17	0.04	0.02
Sample 23 (PIPI)	0.2	0.15	+	0.18	0.43	0.01	0.09
Sample 24 (PIPI)	0.2	0.09	-	0.13	0.43	0.01	0.06

ND = not detected (0.01 mg/kg detection limit).

**Table 8:** Results of LC-MS and rapid test kits for Okadaic Acid in naturally contaminated Pipis [PIPI] Note: Neogen qualitative test (±) with Limit of Quantification = 0.08 mg/kg; Abraxis PP2A Working Range = 0.06–0.35 mg/kg; Beacon ELISA Limit of Quantification = 0.1 mg/kg; Abraxis ELISA Working Range = 0.1–5.0 mg/kg; Europroxima ELISA Limit of Quantification = 0.04 mg/kg.

Sample No. and Shellfish Matrix	OA mg/kg	LC-MS	Neogen	Abraxis PP2A	Beacon ELISA	Abraxis ELISA	Europroxima ELISA
Sample 25 (PIPI)	0.1	0.1	-	0.04	0.07	0.02	0.03
Sample 26 (PIPI)	0.1	0.1	-	0.10	0.05	0.01	0.02
Sample 27 (PIPI)	0.1	0.1	-	0.04	0.06	0.07	0.02
Sample 28 (PIPI)	0.1	0.1	-	0.07	0.10	0.04	0.03
Sample 29 (PIPI)	0.1	0.1	-	0.07	0.06	0.03	0.03
Sample 30 (PIPI)	0.1	0.1	-	0.05	0.08	0.02	0.02
Sample 31 (PIPI)	0.1	0.1	-	0.15	0.06	0.02	0.02
Sample 32 (PIPI)	0.1	0.1	-	0.10	0.06	0.06	0.02
Sample 43 (PIPI)	0.1	0.1	-	0.06	0.10	0.18	0.03
Sample 44 (PIPI)	0.1	0.1	-	NS	0.08	0.17	0.02
Sample 33 (PIPI)	0.2	0.2	+	0.23	0.08	0.25	0.03
Sample 34 (PIPI)	0.2	0.2	+	0.22	0.13	0.14	0.02
Sample 35 (PIPI)	0.2	0.2	+	0.24	0.10	0.24	0.03
Sample 36 (PIPI)	0.2	0.2	+	0.16	0.11	0.17	0.02
Sample 37 (PIPI)	0.2	0.2	+	0.25	0.13	0.22	0.02
Sample 38 (PIPI)	0.2	0.2	+	0.25	0.04	0.14	0.04
Sample 39 (PIPI)	0.2	0.2	+	0.20	0.06	0.06	0.02
Sample 40 (PIPI)	0.2	0.2	+	0.27	0.05	0.16	0.01
Sample 41 (PIPI)	0.2	0.2	+	0.22	0.10	0.05	0.02
Sample 42 (PIPI)	0.2	0.2	+	0.23	0.11	0.02	0.02
Sample 45 (PIPI)	0.3	0.3	+	0.38	0.05	0.21	0.03
Sample 46 (PIPI)	0.3	0.3	+	0.39	0.06	0.19	0.02
Sample 47 (PIPI)	0.3	0.3	+	0.39	0.05	0.33	0.02
Sample 48 (PIPI)	0.3	0.3	+	0.36	0.09	2.05	0.03
Sample 49 (PIPI)	0.3	0.3	+	0.33	0.07	0.88	0.02
Sample 50 (PIPI)	0.3	0.3	+	0.36	0.10	0.11	0.03
Sample 51 (PIPI)	0.3	0.3	+	0.34	0.17	0.23	0.03
Sample 52 (PIPI)	0.3	0.3	+	0.34	0.06	0.24	0.03
Sample 53 (PIPI)	0.3	0.3	+	0.32	0.08	0.19	0.02
Sample 54 (PIPI)	0.3	0.3	+	0.25	0.05	0.17	0.06

NS = no sample.

**Table 9:** List of DST rapid test kits available, their method details and requirements (NR = not reported; ND = not detected). Note: LC-MS Cost ~$300 per sample and ~2 h for analysis. * AU$1 has been added to the cost of each sample for consumables.

Kit No./Name	1. Neogen	2. Abraxis PP2A	3. Beacon ELISA	4. Abraxis ELISA	5. EuroProxima ELISA
Method	Lateral Flow Assay (LFA)—single sample	Protein Phosphatase Inhibition (PPI)—96 well plate	ELISA 96 well plate	ELISA 96 well plate	ELISA 96 well plate
Qualitative or Quantitative	Qualitative	Quantitative	Quantitative	Quantitative	Quantitative
Analogues and Cross reactivity	OA (100%), DTX-1 (89%), DTX-2 (47%) & DTX-3	OA (1.2 nM), DTX-1 (1. 6nM), DTX-2 (1.2 nM), DTX3	OA (100%), DTX-1 (120%), DTX-2 (20%)	OA (100%), DTX-1 (50%), DTX-2 (50%)	OA (100%), DTX-1 (78%), DTX-2 (2.6%)
Limit of Quantification or Working Range	0.08 mg/kg [25]	0.06–0.35 mg/kg	0.1 mg/kg	0.1–5.0 mg/kg	0.04 mg/kg
Standards included	no	0.4, 0.6, 1.0, 1.5 and 2.3 µg/L	0, 0.2, 0.5,1,2, 5 µg/L	0, 0.1, 0.2, 0.5, 1, 2, 5 µg/L	0, 0.2, 0.5, 1.0, 2, 5, 10 µg/L
Hydrolysis step	yes	yes	no	yes	no
Amount of tissue required	2 g	5 g	1 g	1 g	1 g
Samples per kit	24	~35–40 samples	~35–40 samples	~40 samples	~35–40 samples
Cost per kit (AU$)	$974.50	$1277	$849	$848	$999
Cost per sample * (AU$)	$42	$33	$22	$22	$26
Scanner (AU$)	$4000				
Reported False Positives	No false positives compared to ND by LC-MS [25]	14% positive compared to ND by LC-MS [25]	NR	Some false positives [26]	NR
Time for Analysis	~ 1.5 h	~ 3 h	~ 3 h	~ 4 h	~ 3 h

**Table 10:** Summary of results comparing LC-MS (Laboratory 3) and five commercially available test kits to spiked Australian shellfish (results are across all species-specific shellfish matrices). Note: Abraxis PP2A Working Range (WR) = 0.06 to 0.35 mg/kg; Beacon ELISA Limit of Quantification (LOQ) = 0.1 mg/kg; Abraxis ELISA Working Range = 0.1–5.0 mg/kg; Europroxima ELISA Limit of Quantification = 0.04 mg/kg; ML = Maximum limit (=Regulatory Limit 0.2 eq OA mg/kg); Repeatability is defined as the standard deviation of the mean (see Methods).

	LC-MS	Neogen	Abraxis PP2A	Beacon ELISA	Abraxis ELISA	Europroxima ELISA
% False Positive (blank matrix)	0 (0/8)	0 (8/8)	25 (2/8)	0 (0/8)	0 (0/8)	13 (1/8)
% False Negative (spiked matrix)	0 (0/54)	50 (23/46)	-	-	-	-
% Results outside WR or LOQ	-	-	29 (13/45)	43 (20/46)	59 (27/46)	65 (30/46)
% Samples Underestimated	7 (3/46)	-	27 (12/45)	22 (10/46)	24 (11/46)	33 (15/46)
% Samples Equal or Overestimated	93 (43/46)	-	44 (20/45)	35 (16/46)	17 (8/46)	2 (1/46)
% Falsely Compliant with ML (overall)	7 (2/28)	18 (5/28)	29 (8/28)	79 (22/28)	71 (20/28)	100 (28/28)
% Falsely Compliant with ML (spiked)	25 (2/8)	63 (5/8)	88 (7/8)	25 (2/8)	100 (8/8)	100 (8/8)
% Falsely Compliant with ML (naturally contaminated)	0 (0/20)	0 (0/20)	5 (1/20)	100 (20/20)	55 (11/20)	100 (20/20)
% Falsely Non-compliant with ML	0 (54/54)	0 (54/54)	0 (53/53)	0 (54/54)	0 (54/54)	0 (54/54)
Mean (SD) Recovery %	106.5 (22.2)	-	92.2 (34.2)	77.7 (51.2)	66.2 (107.9)	26.7 (29.1)
Repeatability (0.1-0.3 eq OA mg/kg PIPI)	0.00	-	0.01	0.00–0.01	0.02–0.18	0.00
Coefficient of Determination (r^2^)	0.86	-	0.72	0.05	0.08	0.01

**Table 11:** List of Australian shellfish samples, toxin volume of CRM added per 3 g of homogenised shellfish tissue, and OA equivalent concentrations (shaded) dispatched to each laboratory for DST determination using LC-MS.

Matrix	DST Spiking Volumes				Total
	OA Only	DTX-1 Only	DTX-2 Only	OA/DTX-1/DTX-2	
Sydney Rock Oysters	7.2 µL/3 g (3) *	14 µL/3 g	8 µL/3 g	35, 17.6, 16 µL/3 g	6
Pacific Oyster	7.2 µL/3 g	14 µL/3 g	8 µL/3 g	35, 17.6, 16 µL/3 g	4
Mussel	7.2 µL/3 g	14 µL/3 g	8 µL/3 g	7.2, 17.6, 16 µL/3 g	4
Pipi	7.2 µL/3 g	14 µL/3 g	8 µL/3 g	7.2, 17.6, 16 µL/3 g	4
Concentration mg/kg	0.02 mg	0.04 mg	0.01	0.02 or 0.1 ^#^, 0.05, 0.02	
Positive Control (CRM DSP-Mus-c)	-	-	-	-	1
Total Samples					N = 19

* n = 3 for reproducibility/repeatability; ^#^ 0.02 mg/kg for mussel and pipi; 0.1 mg/kg for Sydney Rock Oysters and Pacific Oyster.

## Data Availability

Data is contained within the article.

## Appendix A

**Table A1:** Methods, detection limits, limit of quantification/reporting and measurement uncertainty (standard uncertainty at the LOR) as reported by each laboratory for LC-MS/MS and LM-MS determination of DSTs in shellfish.

	Method	Limit of Detection	Limit of Quantification (LOQ)/Limit of Reporting (LOR)	Measurement Uncertainty
Lab 1 LC-MS/MS	LC-MS/MS Method similar to McNabb (2005) and Villar-Gonzalez et al. (2011) and the EU-Harmonised method from the EU Reference Lab. That is, an 80% MeOH extraction, with two portions of the extract analysed after (1) hexane-cleanup, (2) alkaline hydrolysis (to convert esters to acids).	0.004 mg/kg OA, DTX-1, DTX-2	0.01 mg/kg OA, DTX-1, DTX-2	25% OA 26% DTX-1 24% DTX-2 (at a confidence level of 95%)
Lab 2 LC-MS/MS	Multitoxin LC-MS/MS method for lipophilic toxins based on McNabb 2005 with IANZ (ISO 17025) accreditation.	0.001–0.002 mg/kg OA, DTX-1, DTX-2	0.01 mg/kg OA, DTX-1, DTX-2	21% at 0.01 mg/kg
Lab 3 LC-MS	Sample extraction was performed using the method as described by McNabb et al. (2005). OA analysis was conducted using a Thermo Scientific™ Q EXACTIVE™ high resolution mass-spectrometer equipped with an electrospray ionization. Chromatographic separation was performed on a Thermo Scientific™ ACCELA™ UPLC system.	0.006 mg/kg OA 0.007 mg/kg DTX-1 0.007 mg/kg DTX-2	0.021 mg/kg OA 0.023 mg/kg DTX-1 0.024 mg/kg DTX-2	19% OA 21% DTX-1 12 % DTX-2
Lab 4 LC-MS/MS	LC-MS/MS using the instrument AB ScieX Triple Quad 6500.	~5–10 × lower than the LOQ/LOR	0.025 mg/kg OA, DTX-1 0.015 mg/kg DTX-2	20% Total OA 20% Total DTX-1 20% Total DTX-2 15% Free OA 15% Free DTX-1 10% Free DTX-2

## References

[1] Hallegraeff G., Enevoldsen H., Zingone A. (2021). Global harmful algal bloom status reporting. Harmful Algae.

[2] Lee T.C.-H., Fong F.L.-Y., Ho K.-C., Lee F.W.-F. (2016). The Mechanism of Diarrhetic Shellfish Poisoning Toxin Production in *Prorocentrum* spp.: Physiological and Molecular Perspectives. Toxins.

[3] Reguera B., Riobó P., Rodríguez F., Díaz P.A., Pizarro G., Paz B., Franco J.M., Blanco J. (2014). Dinophysis Toxins: Causative Organisms, Distribution and Fate in Shellfish. Mar. Drugs.

[4] McNabb P., Selwood A.I., Holland P.T., Aasen J., Aune T., Eaglesham G., Hess P., Igarishi M., Quilliam M., Slattery D. (2005). Multiresidue Method for Determination of Algal Toxins in Shellfish: Single-Laboratory Validation and Interlaboratory Study. J. AOAC Int..

[5] Yasumoto T., Oshima Y., Sugawara W., Fukuyo Y., Oguri H., Igarashi T., Fujita N. (1980). Identification of Dinophysis fortii as the causative organism of diarrhetic shellfish poisoning. Bull. Jpn. Soc. Sci. Fish..

[6] Yasumoto T., Oshima Y., Yamaguchi M. (1978). Occurrence of a new type of shellfish poisoning in the Tohoku district. Bull. Jpn. Soc. Sci. Fish..

[7] Lembeye G., Smayda T.J., Shimizu Y. (1993). DSP Outbreak in Chilean Fjords. Toxic Phytoplankton Blooms in the Sea.

[8] Taylor M., McIntyre L., Ritson M., Stone J., Bronson R., Bitzikos O., Rourke W., Galanis E., Team O.I. (2013). Outbreak of Diarrhetic Shellfish Poisoning Associated with Mussels, British Columbia, Canada. Mar. Drugs.

[9] Whyte C., Swan S., Davidson K. (2014). Changing wind patterns linked to unusually high Dinophysis blooms around the Shetland Islands, Scotland. Harmful Algae.

[10] Ajani P., Ingleton T., Pritchard T., Armand L. (2011). Microalgal blooms in the coastal waters of New South Wales, Australia. Proc. Linn. Soc. N. S. W..

[11] Hallegraeff G.M., Lucas I.A.N. (1988). The marine dinoflagellate genus Dinophysis (Dinophyceae)—Photosynthetic, neritic and non-photosynthetic, oceanic species. Phycologia.

[12] McCarthy P.M. (2013). Census of Australian Marine Dinoflagellates. http://www.anbg.gov.au/abrs/Dinoflagellates/index_Dino.html.

[13] Quaine J., Kraa E., Holloway J., White K., McCarthy R., Delpech V., Trent M., McAnulty J. (1997). Outbreak of gastroenteritis linked to eating pipis. N. S. W. Public Health Bull..

[14] Madigan T.L., Lee K.G., Padula D.J., McNabb P., Pointon A.M. (2006). Diarrhetic shellfish poisoning (DSP) toxins in South Australian shellfish. Harmful Algae.

[15] Burgess V., Shaw G. (2001). Pectenotoxins—An issue for public health: A review of their comparative toxicology and metabolism. Environ. Int..

[16] Quilliam M.A., Xie M., Hardstaff W.R. (1995). Rapid Extraction and Cleanup for Liquid Chromatographic Determination of Domoic Acid in Unsalted Seafood. J. AOAC Int..

[17] Christian B., Luckas B. (2007). Determination of marine biotoxins relevant for regulations: From the mouse bioassay to coupled LC-MS methods. Anal. Bioanal. Chem..

[18] Lequin R.M. (2006). Historical background of the invention of EIA and ELISA—Response. Clin. Chem..

[19] Jawaid W., Meneely J.P., Campbell K., Melville K., Holmes S.J., Rice J., Elliott C.T. (2015). Development and Validation of a Lateral Flow Immunoassay for the Rapid Screening of Okadaic Acid and All Dinophysis Toxins from Shellfish Extracts. J. Agric. Food Chem..

[20] Macleod C., Burrell S., Holland P. (2015). Review of the Currently Available Field Methods for Detection of Marine Biotoxins in Shellfish Flesh.

[21] (2015). Codex Alimentarius. Standard for Live and Raw Bivalve Molluscs.

[22] EFSA (2009). Scientific Opinion of the Panel on Contaminants in the Food Chain on a Request from the European Commission on Marine Biotoxins in Shellfish—Pectenotoxin Group. ESFA J..

[23] FSANZ, Maximum Levels of Non-Metal Contaminants F2017C00333 S19-5. In Australia New Zealand Food Standards Code—Schedule 19. https://www.legislation.gov.au/Details/F2016C00197.

[24] AOAC (2019). AOAC International Guidelines for Validation of Qualitative Binary Chemistry Methods. J. AOAC Int..

[25] Turner A.D., Goya A.B. (2016). Comparison of four rapid test kits for the detection of okadaic acid-group toxins in bivalve shellfish from Argentina. Food Control..

[26] Eberhart B.-T.L., Moore L.K., Harrington N., Adams N.G., Borchert J., Trainer V.L. (2013). Screening Tests for the Rapid Detection of Diarrhetic Shellfish Toxins in Washington State. Mar. Drugs.

[27] Farrell H., Ajani P., Murray S., Baker P., Webster G., Brett S., Zammit A. (2018). Diarrhetic Shellfish Toxin Monitoring in Commercial Wild Harvest Bivalve Shellfish in New South Wales, Australia. Toxins.

[28] Suzuki T., Quilliam M. (2011). LC-MS/MS Analysis of Diarrhetic Shellfish Poisoning (DSP) Toxins, Okadaic Acid and Dinophysistoxin Analogues, and Other Lipophilic Toxins. Anal. Sci..

[29] Schirone M., Berti M., Visciano P., Chiumiento F., Migliorati G., Tofalo R., Suzzi G., Di Giacinto F., Ferri N. (2018). Determination of Lipophilic Marine Biotoxins in Mussels Harvested from the Adriatic Sea by LC-MS/MS. Front. Microbiol..

[30] Uchida H., Watanabe R., Matsushima R., Oikawa H., Nagai S., Kamiyama T., Baba K., Miyazono A., Kosaka Y., Kaga S. (2018). Toxin Profiles of Okadaic Acid Analogues and Other Lipophilic Toxins in Dinophysis from Japanese Coastal Waters. Toxins.

[31] European Union (2015). Harmonised Standard Operating Procedure for determination of Lipophilic Marine Biotoxins in Molluscs by LC-MS/MS.

[32] Turner A.D., Tarnovius S., Hatfield R.G., Teixeira-Alves M., Broadwater M., Van Dolah F., Garcia-Mendoza E., Medina D., Salhi M., Goya A.B. (2020). Application of Six Detection Methods for Analysis of Paralytic Shellfish Toxins in Shellfish from Four Regions within Latin America. Mar. Drugs.

[33] Dubois M., Demoulin L., Charlier C., Singh G., Godefroy S.B., Campbell K., Elliott C.T., Delahaut P. (2010). Development of ELISAs for detecting domoic acid, okadaic acid, and saxitoxin and their applicability for the detection of marine toxins in samples collected in Belgium. Food Addit. Contam. Part A Chem. Anal. Control. Expo. Risk Assess..

[34] Smienk H., Domínguez E., Rodríguez-Velasco M.L., Clarke D., Kapp K., Katikou P., Cabado A.G., Otero A., Vieites J.M., Razquin P. (2013). Quantitative Determination of the Okadaic Acid Toxins Group by a Colorimetric Phosphatase Inhibition Assay: Interlaboratory Study. J. AOAC Int..

[35] Smienk H.G.F., Calvo D., Razquin P., Domínguez E., Mata L. (2012). Single Laboratory Validation of A Ready-to-Use Phosphatase Inhibition Assay for Detection of Okadaic Acid Toxins. Toxins.

[36] Johnson S., Harrison K., Turner A.D. (2016). Application of rapid test kits for the determination of Diarrhetic Shellfish Poisoning (DSP) toxins in bivalve molluscs from Great Britain. Toxicon.

[37] Turnbull A.R., Tan J.Y., Ugalde S.C., Hallegraeff G.M., Campbell K., Harwood D.T., Dorantes-Aranda J.J. (2018). Single-Laboratory Validation of the Neogen Qualitative Lateral Flow Immunoassay for the Detection of Paralytic Shellfish Toxins in Mussels and Oysters. J. Aoac Int..

[38] ASQAAC (2016). The Australian Shellfish Quality Assurance Program Operations Manual.

